# Knockdown of PKM2 Suppresses Tumor Growth and Invasion in Lung Adenocarcinoma

**DOI:** 10.3390/ijms161024574

**Published:** 2015-10-15

**Authors:** Hong Sun, Anyou Zhu, Lunjun Zhang, Jie Zhang, Zhengrong Zhong, Fengchao Wang

**Affiliations:** 1 Department of Clinical Laboratory Science, the First Affiliated Hospital of Bengbu Medical College, Bengbu 233004, China; E-Mails: sunhong0222@126.com (H.S.); anyou1968@163.com (A.Z.); zhanglj4723@126.com (L.Z.); zhongzrong@sina.com (Z.Z.); 2 Department of Pathology, Shanghai Chest Hospital, Shanghai Jiaotong University, Shanghai 200030, China; E-Mail: zhangjiechest@126.com

**Keywords:** lung adenocarcinoma, pyruvate kinase M2, glycolysis, fatty acid synthesis, invasion

## Abstract

Accumulating evidence shows that activity of the pyruvate kinase M2 (PKM2) isoform is closely related to tumorigenesis. In this study, we investigated the relationship between*PKM2* expression, tumor invasion, and the prognosis of patients with lung adenocarcinoma. We retrospectively analyzed 65 cases of patients with lung adenocarcinoma who were divided into low and a high expression groups based on *PKM2*immunohistochemical staining. High *PKM2* expression was significantly associated with reduced patient survival. We used small interfering RNA (siRNA) technology to investigate the effect of targeted *PKM2*-knockout on tumor growth at the cellular level. *In vitro*, siRNA-mediated *PKM2*-knockdown significantly inhibited the proliferation, glucose uptake (25%), ATP generation (20%) and fatty acid synthesis of A549 cells, while the mitochondrial respiratory capacity of the cells increased (13%).Western blotting analysis showed that *PKM2*-knockout significantly inhibited the expression of the glucose transporter GLUT1 and ATP citrate lyase, which is critical for fatty acid synthesis. Further Western blotting analysis showed that *PKM2*-knockdown inhibited the expression of matrix metalloproteinase 2 (MMP-2) and vascular endothelial growth factor (VEGF), which are important in degradation of the extracellular matrix and angiogenesis, respectively. These observations show that *PKM2* activates both glycolysis and lipid synthesis, thereby regulating cell proliferation and invasion. This information is important in elucidating the mechanisms by which *PKM2* influences the growth and metastasis of lung adenocarcinoma at the cellular and molecular level, thereby providing the basic data required for the development of *PKM2*-targeted gene therapy.

## 1. Introduction

Unlike most normal cells, glucose metabolism is highly active during tumorigenesis. This particular biological characteristic of the tumor, which is called the “Warburg effect”, is considered to be the seventh characteristic sign of tumors [1]. Aerobic glycolysis, which is a biologically uneconomical mechanism of energy production, is essential to tumor cells for a number of reasons. Although glycolysis generates less ATP than that produced by oxidative phosphorylation (OXPHOS), it can be produced more rapidly by this pathway, which is essential for the needs of rapidly growing tumor cells. In addition, the intermediate products of glycolysis can be used by tumor cells to synthesize the high levels of lipids and proteins required to meet the abnormally high requirement of rapidly growing tumor cells for biological macromolecules [2,3].

The energy generation mechanism of the Warburg effect is more complex under certain conditions. For example, under hypoxic conditions, increased HIF (hypoxia induced factor) expression or abnormal activation of some oncogenes, such as *MYC* and *AKT*, promotes increased expression of glycolytic enzymes, thus enhancing the glycolytic capacity of the cell [4,5,6]. Furthermore, glycolysis activation in turn promotes increased expression of some oncogenes, forming a positive feedback loop between oncogene activation and glycolysis. This positive feedback mechanism results in the malignant transformation of tumor cells, which is associated with enhanced invasion capacity and resistance to chemotherapy [7,8]. Targeting the glycolytic pathway of tumors to break this positive feedback loop is therefore, a promising focus of research into the treatment or tumors and adjuvant therapy [8,9].

Pyruvate kinase (*PK*), which is a key regulator of the Warburg effect, catalyzes the dephosphorylation of phosphoenolpyruvate to pyruvate [10]. There are four *PK* isoenzymes; L, R, M1 and M2. The L and R isoforms are primarily expressed in the liver and red blood cells [11]. The isoenzymes *PKM1* and *PKM2* are originally derived from the *PKM* gene [12]. During embryogenesis, *PKM2* is gradually replaced by *PKM1*. In contrast, *PKM1* is significantly reduced during tumorigenesis, while the expression of *PKM2* increases. Accumulating evidence shows that *PKM2* activity is closely related to the occurrence and development of tumors, with *PKM2* activity being more conducive to the increased needs of tumor cells for the glycolytic intermediates required to promote tumor proliferation and invasion and maintain the malignant phenotype of tumor [13,14].

Recent research has also indicated that *PKM2* upregulation promotes tumor invasion in breast cancer and intestinal cancer [15,16,17]. High invasiveness of tumors is an important factor associated with poor prognosis in cancer patients. Lung adenocarcinoma, which is prone to distant metastasis, is the most common pathological pattern of malignant tumors [18]; however, the association between PKM2 and invasion and prognosis of lung adenocarcinoma remains to be determined. In the present study, we investigated the characteristics of *PKM2* expression as well as the relationship between *PKM2* expression, tumor invasion, and the prognosis of patients with lung adenocarcinoma. This information is important in elucidating the mechanisms by which *PKM2* influences the growth and metastasis of lung adenocarcinoma at the cellular and molecular level, thereby providing the basic data required for the development of *PKM2*-targeted gene therapy.

## 2. Results

### 2.1. Correlation between PKM2 Expression and the Clinicopathological Features of Lung Adenocarcinoma

**Figure 1:**
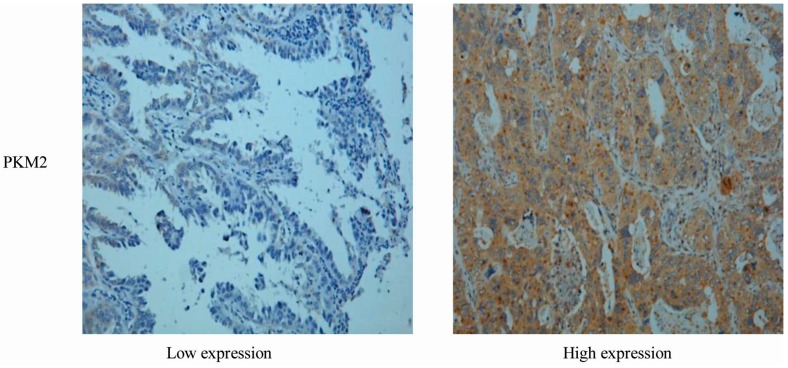
*PKM2* expression in representative tumor tissues (magnification, ×400).

Currently, reports on the correlation between *PKM2* expression in primary tumors and the clinicopathological features of lung adenocarcinoma in primary tumors are rare. In this study, we retrospectively analyzed 65 cases of patients with lung adenocarcinoma (45 males and 20 females; age range, 28–75 years). Patients with lung cancer were divided into a low expression group and a high expression group according to the total PKM2immunohistochemical staining score. Representative examples of low and high *PKM2* expression evaluated by immunohistochemical staining are shown in Figure 1. There was no significant difference in *PKM2* expression between male and female patients.

If 60 years of age was taken as a cut-off, there was also no significant difference between *PKM2* expression and the age of patients. Using a tumor transverse diameter of 3 cm as a cut-off, there was no significant difference in *PKM2* expression between tumors ≤3 cm and tumors >3 cm, while there was a significant difference in *PKM2* expression among tumors with different degrees of differentiation (good, medium and poor). It is noteworthy that there was a very significant difference in *PKM2* expression between pathological stages N0 and N1 and between pathological stages M0 and M1 (Table 1).

**Table 1:** Correlation between *PKM2* expression and the clinicopathological features.

Characteristic	*PKM2* (IHC Score)		
	No. of Patients(*n* = 65)		
	Negative (37)	Positive (28)	*p*-Value
**Age**			
<60 years	18	12	0.643
≥60 years	19	16	
**Gender**			
Male	23	17	0.905
Female	14	11	
**Tumor Differentiation**			
Well	25	10	0.011
Poor	12	18	
**Tumor Size (cm)**			
≤3	25	13	0.087
>3	12	15	
**Pathological N Stage**			
N0	23	8	0.007
N1	14	20	
**Metastasis**			
M0	24	10	0.020
M1	13	18	

**Figure 2:**
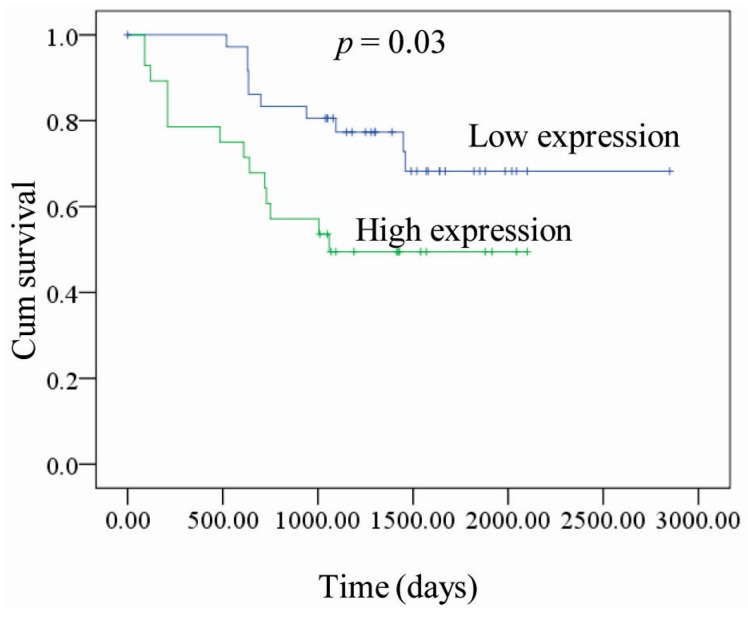
Kaplan-Meier recurrence-free survival curve according to PKM2 expression in patients with lung adenocarcinoma. A total of 65 clinical human lung adenocarcinomas cases were subjected to immunohistochemical analyses with anti-PKM2 antibody. The survival time of PKM2-negative patients is significantly longer than that of PKM2-positive patients.

In order to understand the effect of *PKM2* expression on patient survival, we plotted the survival time of patients with high and low *PKM2* expression (Figure 2). The resulting survival curve showed that the survival time of patients with high *PKM2* expression was significantly shortened compared with that of the patients with low *PKM2* expression (*p* = 0.03).

### 2.2. Tumor Growth Was Inhibited by PKM2 Gene Silencing in A549 Cells

**Figure 3:**
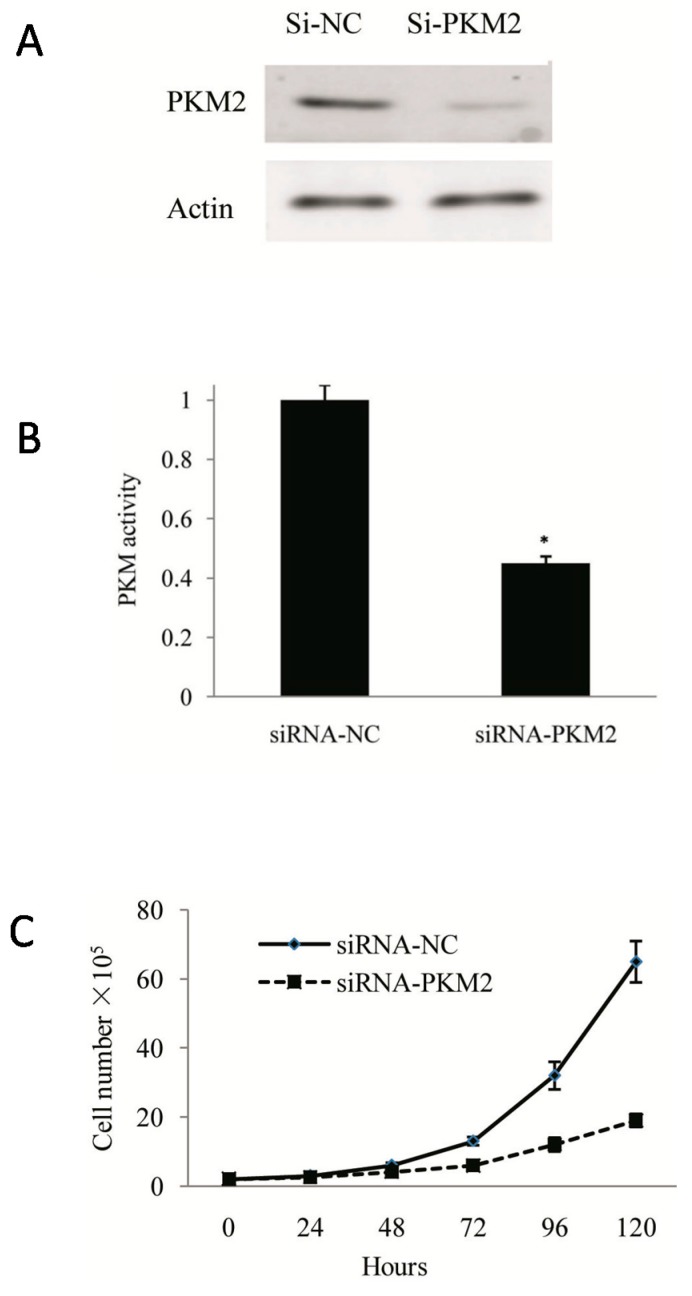
Effect of knockdown of *PKM2* on proliferation of A549 cells. (**A**) PKM2 protein expression in si-NC and si-*PKM2* A549 cells was detected by Western Blotting to evaluate the efficiency of *PKM2*-knockdown at 72 h post-transfection; (**B**) *PKM2* activity in si-*PKM2* A549 cells, expressed as percentage of the enzyme activity in parental A549 cells at 72 h post-transfection; (**C**) Cell viability was determined by counting si-*PKM2* A549 cells. * *p* < 0.05. The data shown are representative of three separate experiments.

High expression of *PKM2* is related to high invasion and poor prognosis in lung adenocarcinoma; thus, we hypothesized that targeted inhibition of *PKM2* can be used effectively to inhibit tumor growth. In this study, we used small interfering RNA (siRNA) technology to investigate the effect of targeted *PKM2*-knockout on tumor growth at the cellular level. After siRNA-*PKM2* transfection for 72 h, intracellular *PKM2* expression was shown to be significantly inhibited by Western blot analysis (Figure 3A). Furthermore, PK activity was significantly decreased in siRNA-*PKM2* cells compared with that in the cells of the control group (*p* < 0.05) (Figure 3B). In order to understand the effects of inhibiting *PKM2* expression on tumor growth, we further assessed the proliferative capacity of the cells in each group by cell counting. The results showed that the proliferation rate in the siRNA-*PKM2* group was significantly reduced compared with that in the siRNA-NC control group (*p* < 0.05) (Figure 3C).

### 2.3. PKM2-Knockdown Inhibited the Warburg Effect in Tumors

**Figure 4:**
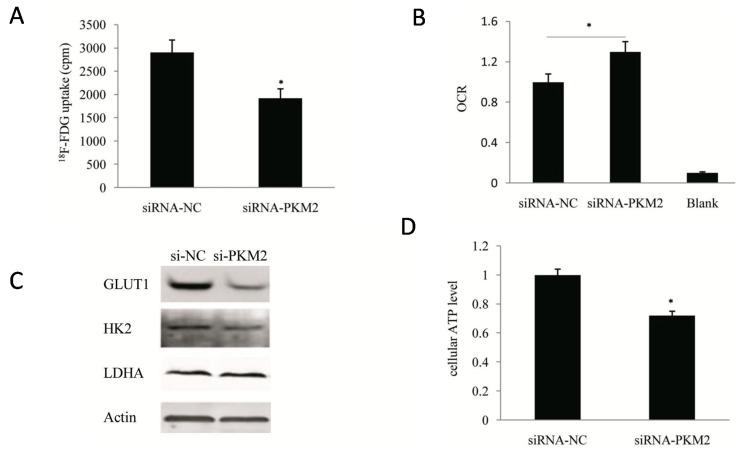
Inhibition of the Warburg effect in A549 cell lines by si-*PKM2*. (**A**) Comparison of glucose uptake between si-*PKM2* A549 cells and control si-NC A549 cells. At 72h post-transfection, A549 cells were incubated in DMEM with ^18^F-FDG for 1 h and uptake radioactivity was measured using the well γ-counter; (**B**) Oxygen consumption of A549 cells was determined by the use of the seahorse at 72 h post-transfection with siRNA; (**C**) Influence of *PKM2* siRNA on glycolytic enzymes in A549 cells; (**D**) Quantification of ATP levels in si-NC and si-*PKM2* A549 cells using a luciferin/luciferase-based assay at 72 h post-transfection. * *p* < 0.05. The data shown are representative of three separate experiments.

*PKM2* is a terminase that plays a critical role in glucose metabolism, and its knockout inhibits tumor growth. Thus, clarification of the effect of inhibition of *PKM2* on tumor metabolism, especially on the Warburg effect, is an important focus of our research. Following siRNA-mediated knockdown of *PKM2*, the glucose uptake capacity of A549 cells decreased by 25% compared with the control cells (*p* < 0.05) (Figure 4A), while detection of the oxygen consumption rate (OCR) revealed that *PKM2*-silencing resulted in a 13% increase in the mitochondrial respiratory capacity of the cells (*p* < 0.05) (Figure 4B). These observations confirmed that *PKM2*-silencing significantly inhibited the Warburg effect in tumors. Further analysis of the protein expression levels of the key enzymes in glucose metabolism by Western blotting (Figure 4C) showed that *PKM2*-knockout significantly inhibited the expression of the glucose transporter *GLUT1*. In contrast, the inhibitory effects on the expression of HK2 and LDHA proteins were not significant. Although the molecular mechanism is unclear at present, the reduced glucose uptake capacity of tumor cells following *PKM2*-knockout is clearly related to its inhibitory effect on the expression of GLUT1 protein.

Because the tumor cells are heavily dependent on glycolysis for energy production, significant inhibition of glycolysis may reduce ATP generation in tumor cells. Compared with the control cells, the ATP generation of *PKM2*-silenced A549 cells was decreased by 20% (*p* < 0.05) (Figure 4D).

### 2.4. PKM2-Knockdown Inhibited Fatty Acid Synthesis of Glucose Carbon Source in Tumor Cells

**Figure 5:**
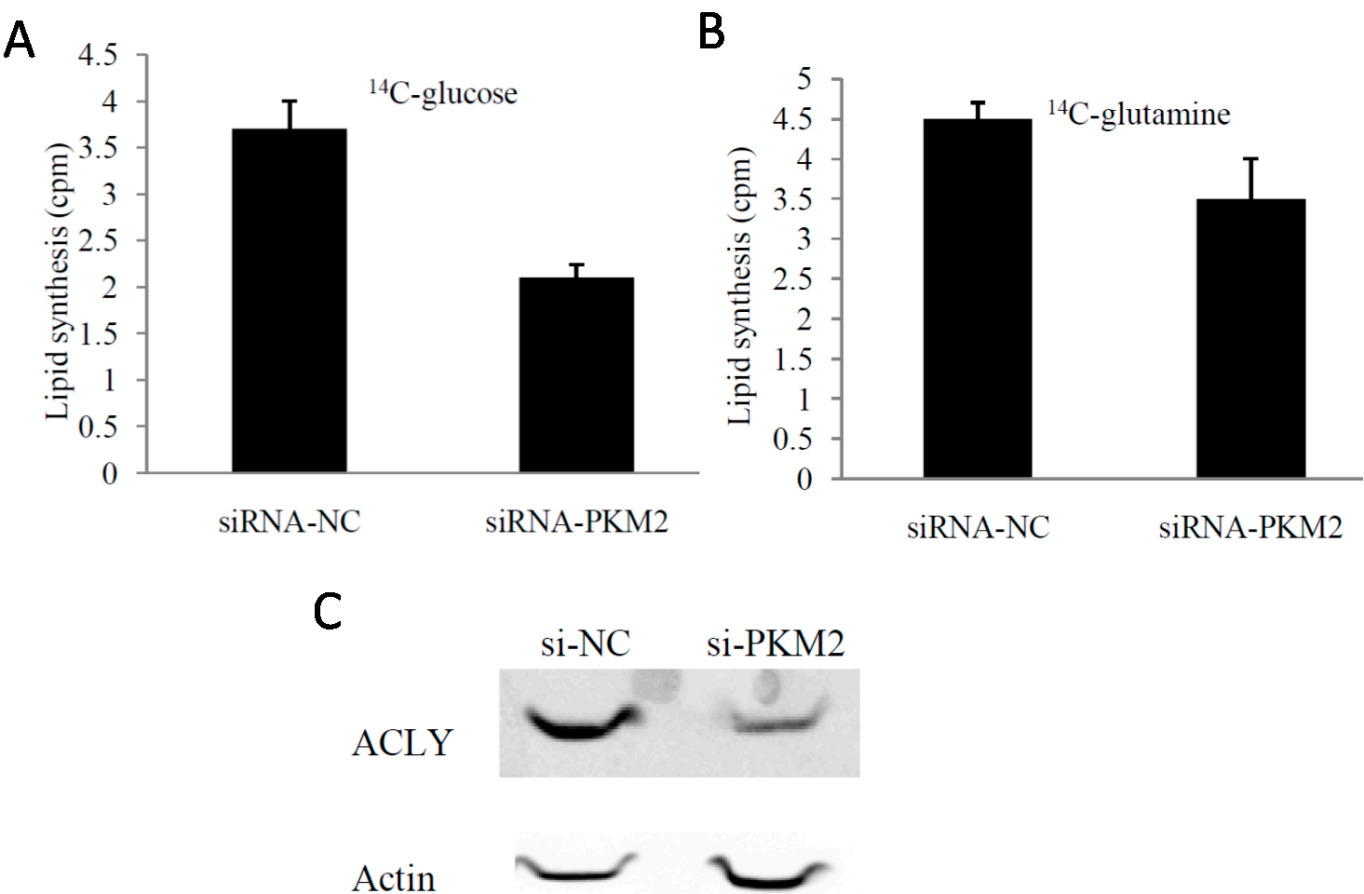
Effects of *PKM2*-knockdown by transient RNAi transfection on lipid synthesis. (**A**) PKM2-knockdown inhibited the glucose-dependent lipid synthesis; (**B**) *PKM2*-knockdown inhibited glutamine-dependent lipid synthesis; (**C**) The expression of *ACLY* was significantly decreased after knockdown of *PKM2* by Western blot analysis.* *p* < 0.05. The data shown are representative of three separate experiments.

The Warburg effect in tumor cells not only provides sufficient ATP for tumor proliferation, but also serves to fulfill the high requirement for biological macromolecules. To determine the effects of *PKM2*-knockout on glucose metabolism and fatty acid synthesis in tumors we used ^14^C-glucose and ^14^C-glutamineas a carbon source. We found that ^14^C-lipid synthesis significantly reduced in *PKM2*-knockout cells (*p* < 0.05) (Figure 5A,B).

ATP citrate lyase (ACLY) catalyzes the conversion of citric acid into acetyl coenzyme A that can be used for fatty acid synthesis. Thus, we investigated the role of ACLY in the inhibitory effect of *PKM2*-knockdown on fatty acid synthesis. We measured the expression of *ACLY* in A549 cells after transfection with siRNA-*PKM*2 for 72 h. Compared with control cells, the *PKM2*-knockout cells showed reduced expression of *ACLY* (Figure 5C). We further demonstrated that the impact of *PKM2* on fatty acid synthesis was mediated by its effects on *ACLY* expression.

### 2.5. Effect of PKM2 Downregulationon the A549 Cell Invasiveness

**Figure 6:**
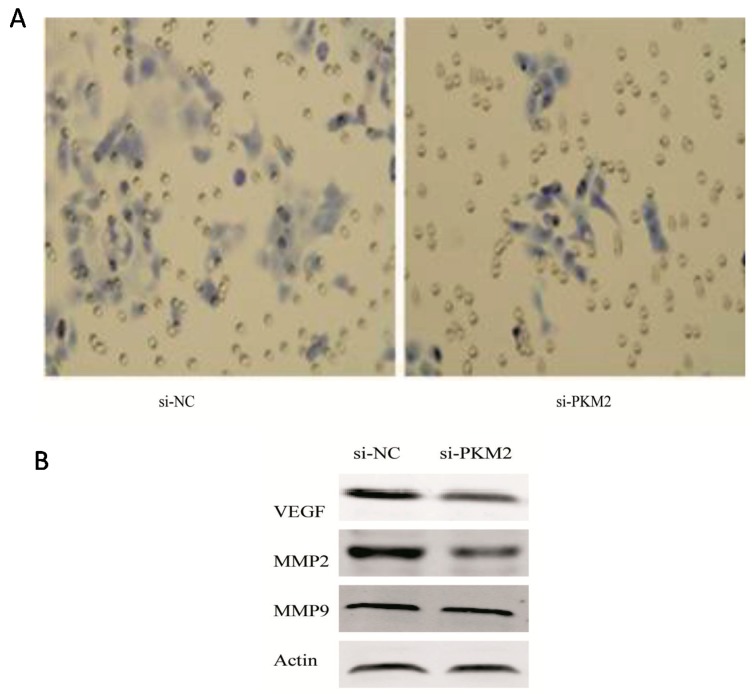
The functional role of *PKM2* in invasion of A549 *in vitro*. (**A**) A549 cells transfected with si-*PKM2* were examined for their invasive capability. At 72 h post-transfection, the number of migrating cells was significantly decreased compared with the control cells (magnification, ×200); (**B**) Effects of *PKM2* knockdown on the expression of *VEGF* (vascular endothelial growth factor), *MMP-2* and *MMP-9* at 72 h post-transfection.

The retrospective clinical study suggests that *PKM2* is closely related to tumor invasion. Furthermore, *PKM2* inhibits ATP and lipid generation by lung adenocarcinoma A549 cells at the cellular level; these two metabolites are closely related to tumor invasion. Therefore, we addressed the functional role of *PKM2* in invasion of cells into the surrounding tissue. Treatment of cells with A549-si*PKM2* cells effectively suppressed cell invasion (Figure 6A). For instance, at 72 h after transfection with siRNA-*PKM2*, the number of A549 cells able to migrate through the filter decreased to 57.4% compared to the cells transfected with control siRNAs.

To further understand the mechanisms by which *PKM2* functions in A549 cell invasion and to determine the events downstream of *PKM2* that are involved in regulation of cell invasion, we examined the expression of proteins known to be involved in the regulation of cell invasiveness was investigated by Western blot. Compared with siRNA-NC-transfected A549 cells, matrix metalloproteinase 2 (*MMP-2*), *VEGF* expression was inhibited in A549-si*PKM2* cells, while matrix metalloproteinase 9 (*MMP-9*) expression was not significantly changed (Figure 6B). These results showed that the invasive capability of A549 cells was significantly decreased after siRNA-mediated *PKM2*-silencing. These observations suggested that *PKM2* plays an important role in the invasion potential of A549 cells.

### 2.6. Discussion

Pyruvate kinase is a key enzyme in the glycolytic pathway that catalyze its substrate phosphoenolpyruvate into pyruvate. Accumulating evidence indicates that the type II isoenzyme *PKM2* is closely associated with the occurrence and development of tumors [19]. In the present study, we investigated the characteristics of *PKM2* expression as well as the relationship between *PKM2* expression, tumor invasion, and the prognosis of patients with lung adenocarcinoma and in solid tumor tissues. We confirmed that compared with the patients with low expression of *PKM2*, high expression of *PKM2* was more likely to produce lymph node metastasis and distant metastasis (*p* < 0.05). Because tumor metabolic activity were closely related to patient prognosis [20,21,22], we further analyzed the survival curves and found that the survival rate of patients with high *PKM2* expression was significantly lower than that of patients with low *PKM2* expression (*p* = 0.03). These results suggest that *PKM2* overexpression is related to poor prognosis in patients with in lung adenocarcinoma; thus, implicating *PKM2* as a potential target for cancer therapy.

We and others have demonstrated that targeted inhibition of *PKM2* inhibits the proliferation of tumors [23,24]. Moreover, we have shown that siRNA-mediated *PKM2*-knockdown also significantly inhibits the invasive potential of lung adenocarcinoma A549 cells, which is also consistent with our clinical observations. The Warburg effect of tumor cells not only generates sufficient ATP, but also to meets the increased demand for carbon units to synthesize fatty acids and other vital substances to maintain their own malignant behavior.

Glycolysis is an important mechanism by which for tumor cells obtain energy. We showed that siRNA-mediated knockdown of *PKM2* significantly inhibited the glucose uptake capacity of A549 cells and ATP generation. In addition, *PKM2*-knockout significantly inhibited the synthesis of fatty acids in tumor cells. It can be speculated that this effect is related to the decreased production of ATP. However, we also demonstrated that *PKM2*-knockdown also significantly inhibited the expression of *ACLY*, which indicated that *ACLY* is also involved in the mechanism by which *PKM2*-knockdown inhibits fatty acid synthesis. Some studies have also demonstrated that *ACLY* is also a cancer gene associated with metabolism [25,26]. Its activation not only promotes the tumor cell proliferation, but also enhances tumor invasiveness. Therefore, the inhibition of *ACLY* by *PKM2*-knockdown may also be involved in the inhibition of the growth and invasiveness of A549 cells. This hypothesis requires confirmation and further investigation of the mechanism by which *PKM2* regulates the expression of *ACLY* protein.

This study also showed that *PKM2*-knockdown decreased the expression of *MMP-2* and *VEGF*, suggesting that *PKM2* is involved in regulating the expression of *MMP-2*, and *VEGF*. These results imply that *PKM2* regulates multiple processes in A549 invasion. It can be hypothesized that *PKM2* induces *MMP-2* production resulting in degradation of the extracellular matrix, while *PKM2* may regulate the A549 invasive capability by affecting angiogenesis through *VEGF*. This hypothesis requires investigation in further studies of the effects of *PKM2* on the expression of tumor invasion-related proteins.

In summary, we show that *PKM2* plays an important role in metastasis at the clinical level as well as in A549 tumor growth at the molecular level. At the metabolic level, *PKM2* activates both glycolysis and lipid synthesis, thereby regulating cell proliferation and invasion. Furthermore, our observations indicate that *PKM2* induces the production of *MMP-2*, which may represent an important mechanism by which *PKM2* is involved in A549 metastasis. These data indicate the potential of *PKM2* in the development of targeted gene therapy of lung adenocarcinoma.

## 3. Experimental Section

### 3.1. Study Population

This was a retrospective study of all patients who were confirmed to have non-small cell lung cancer (NSCLC) based on histopathological finding and underwent surgery after ^18^F-FDG PET/CT between December 2006 and December 2009 at Shanghai Chest Hospital. Our inclusive study population included 65 patients (45 male and 20 female) with a median age of 60 (range, 28–75 years). The Human Investigation Ethical Committee of the First Affiliated Hospital of Bengbu Medical College and Shanghai Chest Hospital approved this study. All procedures involving human specimens were performed with written informed consent according to the Declaration of Helsinki.

### 3.2. Immunohistochemical Staining

Immunohistochemical analyses were performed on paraffin-embedded lung cancer tissues. After microtome sectioning (4 µm slices), the slides were processed for staining. *PKM2* antibodies were purchased from Abcam (Cambridge, UK). The expression of each marker protein was examined and evaluated according to the original protocol reported previously. The slides were scored for intensity of staining (0 to 3) and the percentages of cells with scores of 0 (0%), 1 (1% to 9%), 2 (10% to 49%), and 3 (50% to 100%) were determined. The immunohistochemistry (IHC) score (0 to 9) was defined as the product of the intensity and percentage of cells. Protein expression was judged as positive when the IHC score was greater than or equal to 4. All IHC results were evaluated by two experienced observers who were blind to the condition of the patients. Where discrepancies occurred between two readers, the two readers reached a consensus.

### 3.3. Cell Culture and Viability Assay

A549 cells (human lung adenocarcinoma cell line) were obtained from the Chinese Academy of Sciences and grown in Dulbecco’s modified Eagle’s medium (GIBCO, Carisbad, CA, USA) supplemented with 10% fetal bovine serum (GIBCO), 100 mg/mL penicillin, and 100 mg/mL streptomycin sulfate (GIBCO) at 37 °C and 5% CO_2_. Lipofectamine 2000 (Invitrogen, Carisbad, CA, USA) was used in transient transfection according to the manufacturer’s protocol. The *PKM2*-siRNA and control was purchased from pharma (Shanghai, China). The sequence of si-PKM2 is 5′-CAUCUACCACUUGCAAUUA-3′.

The cell survival rate was examined using Cell Counting Kit-8 (Dojindo Molecular Technologies, Rockville, MD, USA). Cells were plated in 96-well plates at 6000 cells/well. After 24 h of culture, transfection was performed using siRNA according to manufacturer’s protocol. After incubation for 48–72 h, 10 μL CCK-8 was added to each well, and cells were further incubated for 1 h. Absorbance was read at 450 nm using an enzyme micro-plate reader. The IC50 value was calculated using GraphPad Prism version 5.0 software [27]. All experiments were performed in independent triplicate experiments.

### 3.4. Cell Metabolism Assay

Relative cellular ATP content was measured using the ATP bioluminescent somatic cell assay kit (Sigma, St. Louis, MO, USA) according to the manufacturer’s instructions. Briefly, 2 × 10^5^ cells were seeded in a 24-well plate. After 24 h, Cell were washed, centrifuged, and lysed. Lysates were collected, and luminescence was measured using a luminescence reader and normalized to the protein concentration. Measurements were performed in triplicate.

^18^F-FDG cellular uptake can evaluate glucose uptake capacity of tumor cells. Cells were cultured in 12-well cell culture plates, then detached, washed twice, and subsequently incubated in 500 µL of DMEM containing 4 µCi/mL of ^18^F-FDG for 1 h at 37 °C. Pellets were then washed twice with ice-cold PBS. Lysates were produced using 500 µL of 0.1 M NaOH and then the radioactivity of the whole-cell lysates was assayed using a well γ-counter. These readouts were normalized to corresponding protein amounts (Beyotime, Shanghai, China). All experiments were performed in independent triplicate experiments.

Oxygen consumption rate (OCR) was measured using the Seahorse XF24 analyzer (Seahorse Bioscience, Boston, MA, USA). Cells were seeded in a 24-well cell culture microplate and allowed to attach overnight. Approximately 30 min prior to the assay, culture medium was changed to the Seahorse assay medium, DMEM containing 5 mM glucose or 2 mM l-glutamine, and OCR was measured according to the manufacturer’s instructions.

### 3.5. Cell Invasion Assays

Cell invasion was assessed using Transwell chambers (Corning, Acton, MA, USA) according to the manufacturer’s instructions. Briefly, 2 × 10^4^ cells pre-starved in serum-free Dulbecco’s modified Eagle’s medium for 12 h were placed into the upper chamber with 0.2 mL of serum-free Dul-becco’s modified Eagle’s medium. Dulbecco’s modified Eagle’s medium (0.8 mL) supplemented with 10% fetal bovine serum was placed in the lower chamber as a chemo-attractant. Cells migrating across the membrane were stained using Giemsa stain (Sigma-Aldrich, St. Louis, MO, USA) and counted. All experiments were performed in independent triplicate experiments.

### 3.6. Lipid Synthesis

Samples were assayed for lipid synthesis through ^14^C-glucose or^14^C-gultamine.To begin the assay, 4 µCi/mL ^14^C-glucose or ^14^C-gultamine was added to the cells, and cells were incubated at 37 °C for 6 h. For adherent cells grown on 12-well plate, lipids were extracted by the addition of 500 µL of a hexane:isopropanol solution (3:2 ratio). Wells were washed with an additional 500 µL ofhexane:isopropanol solution, and extracts were combined and dried under N_2_, resuspended in 50 µL of chloroform, and counted in ScintiVerse (Fisher, Pittsburgh, PA, USA) on a Beckman LS 6500 scintillation counter (Beckman Coulter, Brea, CA, USA). Protein concentration was determined by bicinchoninic acid (BCA) assay.

### 3.7. Western Blotting

The proteins were fractionated using SDS/PAGE. Then proteins were transferred onto nitrocellulose membrane for Western blot as described earlier. The membranes were incubated about 3 h at 37 °C with the primary antibodies in PBS with 5% non-fat milk. The primary antibodies were utilized: anti-PKM2 rabbit antibody (1:500, Abcam, Cambridge, UK); anti-ACLY rabbit antibody (1:1000, Proteintech, Wuhan, China); anti-VEGF rabbit antibody (1:1000, Abcam); anti-MMP2 rabbit antibody (1:1000, Abcam); anti-MMP9 rabbit antibody (1:1000, Abcam); anti-β-actin antibody (1:2000, Sigma). Membranes were extensively washed with PBS and incubated with secondary anti-rabbit antibody (1:10,000, LI-COR Biosciences, LincoIn, NE, USA). After washes with PBS, protein bands were scanned and analyzed using a Gel Doc XR system (Bio-Rad, Philadelphia, PA, USA), and analyzed using IMAGE LABTM software version 2.0 (Bio-Rad, Philadelphia, PA, USA).

### 3.8. Statistical Analysis

Continuous variables were analyzed by the Student’s *t*-test, and the results were expressed as mean ± standard deviation. Dichotomous variables were analyzed by the χ^2^ test. To explore the association between recurrence-free survival and PKM2 expression, a Kaplan-Meier survival analysis was performed. Two-sided *p* values of less than 0.05 were considered to be statistically significant. All analyses were performed using SPSS software, version 16.0 (SPSS Inc., Chicago, IL, USA).

## 4. Conclusions

*PKM2* activates both glycolysis and lipid synthesis, thereby regulating cell proliferation and invasion. This information is important in elucidating the mechanisms by which *PKM2* influences the growth and metastasis of lung adenocarcinoma at the cellular and molecular level, thereby providing the basic data required for the development of *PKM2*-targeted gene therapy.
